# Contribution of irrigation to the production of maize, wheat, and rice in the major global producing countries

**DOI:** 10.1093/nsr/nwae374

**Published:** 2024-10-22

**Authors:** Zhipin Ai, Julien Boulange, Xin Zhao, Fadong Li, Rashid Mahmood, Kiril Manevski, Yonghui Yang, Guirui Yu

**Affiliations:** Key Laboratory of Ecosystem Network Observation and Modeling, Institute of Geographic Sciences and Natural Resources Research, Chinese Academy of Sciences, China; College of Resources and Environment, University of Chinese Academy of Sciences, China; United Graduate School of Agricultural Science, Tokyo University of Agriculture and Technology, Japan; Earth System Division, National Institute for Environmental Studies, Japan; Key Laboratory of Ecosystem Network Observation and Modeling, Institute of Geographic Sciences and Natural Resources Research, Chinese Academy of Sciences, China; College of Resources and Environment, University of Chinese Academy of Sciences, China; Water Engineering and Management, Asian Institute of Technology, Thailand; Sino-Danish College, University of the Chinese Academy of Sciences, China; Department of Agroecology, Aarhus University, Denmark; Center for Agricultural Resources Research, Institute of Genetics and Developmental Biology, Chinese Academy of Sciences, China; Center for Agricultural Resources Research, Institute of Genetics and Developmental Biology, Chinese Academy of Sciences, China; Key Laboratory of Ecosystem Network Observation and Modeling, Institute of Geographic Sciences and Natural Resources Research, Chinese Academy of Sciences, China; College of Resources and Environment, University of Chinese Academy of Sciences, China

## Abstract

This study offers new insights into the heterogeneity behind the widely accepted notion that irrigated crops contribute 40% to global food production. It also highlights the potential of irrigation to mitigate the negative effects of climate change on crop yields.

Global megatrends in population growth and climate change, together with regional conflicts, are driving an increasing and unequal demand for food and consequently pose a serious threat to food security worldwide. Studies have shown that increasing cropland productivity, rather than expanding cropland area, may be a more viable strategy to promote food security [1]. From an agronomic perspective, irrigation can greatly enhance crop yield and productivity, particularly in arid regions and during dry seasons [2]. Therefore, irrigation is crucial for improving global crop production and ensuring global food security.

Although it is generally acknowledged that irrigated crops constitute ∼40% of the global crop production [2–4], the exact global contribution remains uncertain. In particular, the rationale, as well as crop- and country-specific variations, have not been fully explained. To our knowledge, Postel [5] provided the first quantitative estimation of the contribution of irrigation to global crop production; based on a comprehensive analysis of national statistics, this pioneer study estimated that irrigated crops constituted ∼36% of global crop production. The development of process-based crop models has enabled more detailed study of irrigation's contribution to the production of different crops. For example, Siebert and Döll [6], as possibly the first researchers to account for crop-specific irrigation contributions via spatially explicit simulation, showed that irrigation contributed ∼26%, 37%, and 77% of the global production of maize, wheat, and rice, respectively. Ai and Hanasaki [7] more recently reported comparable estimates of ∼27%, 30%, and 61%, respectively. Despite enhancement of our understanding of the role of irrigation in the production of different crops at the global scale, there remains a gap in knowledge at the national scale, particularly for the major crop-producing countries.

In this study, we systematically quantified the contribution of irrigation to the national production of different crops. Specifically, we estimated the production of three major crops (maize, wheat, and rice) in the major producing countries under irrigation and rainfed conditions using a global hydrological model with an enhanced crop yield simulation function driven by the latest meteorological data from Inter-Sectoral Impact Model Intercomparison Project (ISIMIP) 3 (see Supplementary file for details). We selected the top 20 producing countries for each crop (Table S1), which produced a combined 88%, 86%, and 93% (averages for 1986–2015) of global maize, wheat, and rice production, respectively [7]. Detailed data on yield, harvest area, and production are presented in Tables S2–S4.

For maize (Fig. 1a), the results show that the contribution of irrigation to production exceeded 40% in four of the top 20 countries (Egypt, Italy, China, and France) and that it fell within 20%–40% in 3 countries (India, Mexico, and United States), and was <20% in the remaining 13 countries. Regarding wheat (Fig. 1b), irrigation contributed to >40% of production in 5 countries (Egypt, Pakistan, India, China, and Iran), but <20% of production in the remaining 15 countries. In the case of rice (Fig. 1c), the contribution of irrigation to production exceeded 40% in 15 countries, it fell within 20%–40% in 3 countries (Myanmar, Brazil, and Nepal), and was <20% in 2 countries (Cambodia and Nigeria).

**Figure 1.**
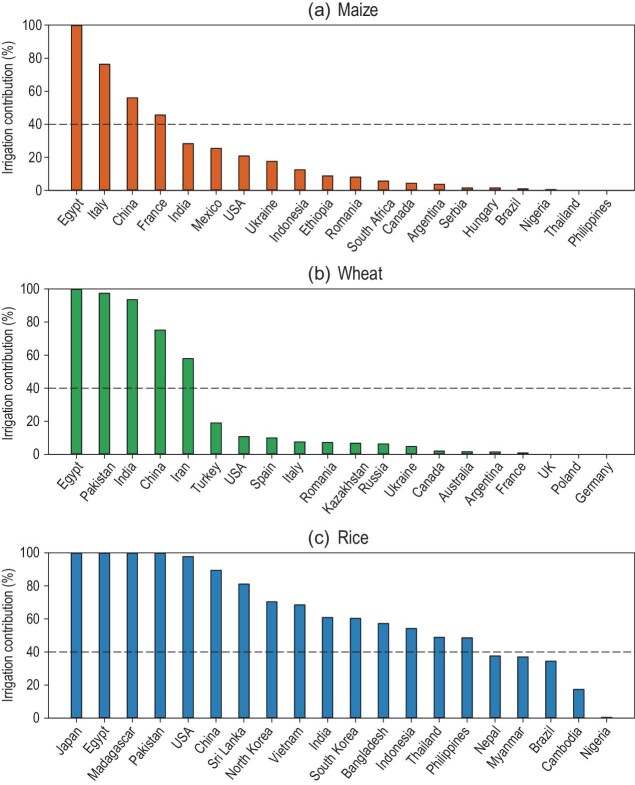
Contribution of irrigation (%) to the production of maize (a), wheat (b), and rice (c) in the top 20 producing countries for each crop.

Among the top 20 countries for the production of each crop, China, India, and Egypt consistently emerged as nations with irrigated production making up >25% of the total production of all three crops. Particularly in China and Egypt, the contribution of irrigation surpassed 50% for all three crops. This finding implies that irrigation is crucial to securing food production in these three countries. Moreover, it highlights the potential for combating food security in other countries through increasing irrigation area and efficiency.

Next, we sought to gain insights into the role of irrigation in climate change adaptation. A previous comprehensive study predicted a 24.1% decrease in maize yield by the end of this century under a high-emissions scenario [8]. Therefore, we analyzed the potential for irrigation to sustain the maize yield under the same high-emissions scenario. In general agreement with the previous results [8], our findings showed a 13% decrease in the average maize yield of the top 20 maize-producing countries in the future under the assumption that the irrigation area remained the same as at present (Fig. S1). Notably, our prediction showed a positive effect of climate change on the maize yield in Canada.

To elucidate the possible effects of irrigation area expansion on crop yield, we performed a sensitivity analysis of the change in maize yield relative to an increase in irrigated maize area. Increasing the irrigation area over currently rainfed cropland had a positive impact on the change in maize yield (Fig. S1). When 25% of rainfed cropland was converted into irrigated cropland per country, the average maize yield change among the top 20 countries was ∼1%, indicating that the negative impacts of climate change on maize yield could generally be avoided under this scenario. Larger yield increments with increasing irrigation area were observed in countries with relatively large yield differences between irrigated and rainfed conditions and relatively low proportions of irrigated cropland (e.g. France, Ukraine, Romania, Canada, Hungary, and Serbia). This further implies that expanding irrigation has the potential to offset the negative impacts of climate change on maize yield and global maize production.

Here, irrigation expansion was assumed to be achieved by converting currently rainfed cropland; however, this theoretical estimation did not consider various factors. For instance, a comprehensive assessment of the socioeconomic and environmental feasibility of irrigation cropland expansion is required, because numerous factors are involved in the design and operation of irrigation, including irrigation water availability, transboundary water allocation, food demand, trade systems, social acceptance, and economic development [9,10].

To our knowledge, our findings are the first to report the contributions of irrigation to the production of maize, wheat, and rice in each major producing country in the world. The results highlight the important role of irrigation in maintaining maize, wheat, and rice production in countries such as China, India, and Egypt. Our study provides new insights into the heterogeneity underlying the common understanding that irrigated crops constitute a 40% contribution to global food production. Specifically, irrigation contributes <40% of national maize and wheat production in at least two-thirds of the top 20 producing countries, yet contributes >40% of national rice production in at least two-thirds of the top 20 producing countries. We also identified the potential for irrigation to offset the negative impacts of climate change on crop production, in particular for countries with larger yield differences between irrigated versus rainfed crops and lower fractions of irrigated cropland.

## Supplementary Material

nwae374_Supplemental_File

## Data Availability

The meteorological datasets used in this study are available at https://data.isimip.org/search/.

## CODE AVAILABILITY

The code of the H08 model is available from the website at https://zenodo.org/records/7344809.
