# Supplementary material for: Contribution of irrigation to the production of maize, wheat, and rice in the major global producing countries
Source: Natl Sci Rev. 2024 Oct 22;11(11):nwae374. doi: 10.1093/nsr/nwae374 (PMC11565227; doi:10.1093/nsr/nwae374)
Supplement: nwae374_Supplemental_File [file nwae374_supplemental_file.docx]

Supplementary file for

**Contribution of irrigation to the production of maize, wheat, and rice in the major global producing countries**

Zhipin Ai^1,2^, Julien Boulange^3^, Xin Zhao^4^, Fadong Li^1,2^, Rashid Mahmood^5^, Kiril Manevski^6,7,8^, Yonghui Yang^8^, Guirui Yu^1,2^

^1^Key Laboratory of Ecosystem Network Observation and Modeling, Institute of Geographic Sciences and Natural Resources Research, Chinese Academy of Sciences, Beijing, China, 100101

^2^College of Resources and Environment, University of Chinese Academy of Sciences, Beijing, China, 101408

^3^United Graduate School of Agricultural Science, Tokyo University of Agriculture and Technology, Tokyo, Japan, 183-8509

^4^Earth System Division, National Institute for Environmental Studies, Tsukuba, Japan, 305-8506

^5^Water Engineering and Management, Asian Institute of Technology, Pathumthani, Thailand, 12120

^6^Sino-Danish College, University of the Chinese Academy of Sciences, Beijing, China, 100049

^7^Department of Agroecology, Aarhus University, Tjele, Denmark, 8830

^8^Center for Agricultural Resources Research, Institute of Genetics and Developmental Biology, Chinese Academy of Sciences, Shijiazhuang, China, 050021

**Contents of this file**

Materials and Methods

Figures S1

Tables S1–S4

References

**Materials and Methods**

**Model H08**

H08 is a global hydrological model that can simulate natural and anthropogenic hydrological processes at daily time scale. The model was initially developed with six sub-models, including land surface, river routing, crop growth, reservoir operation, environmental flow estimation, and anthropogenic water withdrawal [1]. It was enhanced with new schemes on groundwater recharge and abstraction, aqueduct water transfer, local reservoirs, seawater desalination, and return flow and delivery loss [2]. Detailed model descriptions and assessments of the model outputs (e.g., streamflow and crop yield) can be found in a series of previous studies [1–7].

The crop sub-model can accumulate plant biomass at a daily interval until physiological maturity. Biomass estimation is based on crop specific radiation use efficiency and photosynthetic active radiation. Phenological development estimation is based on daily heat unit accumulation theory. The harvest index is used to partition the total aboveground biomass with respect to grain yield. Factors including water and air temperature are used to adjust the yield variation [1]. Recently, through parameter calibration and algorithm improvement, the crop sub-model was enhanced, and the simulated yield have been well evaluated with the FAO statistics (1986–2015) [7].

**Meteorological data**

To simulate the irrigation contribution to crop production, the bias-corrected ISIMIP 3a GSWP3-W5E5 global meteorological data (1980–2015) were used. To clarify the role of irrigation expansion in improving crop yield under climate change, the ISIMIP 3b meteorological data from five general circulation models (GCMs): GFDL-ESM4, UKESM1-0-LL, MPI-EWM1-2-HR, IPSL-CM6A-LR, and MRI-ESM2-0 under SSP5-RCP85 were used (2065–2099). We used the average values from the results driven by the five GCMs. For each dataset, eight daily meteorological variables, including air temperature, wind speed, air pressure, specific humidity, rainfall, snowfall, downward shortwave radiation, and downward longwave radiation were used to run the model. All the data are available from <https://data.isimip.org/search/>.

**Simulation and data processing**

We ran the crop sub-model for maize, wheat, and rice under both rainfed and irrigation conditions for the two periods (1986–2015 and 2070–2099) on a daily scale (detailed information on model setup and simulation is available from <https://zenodo.org/records/13855334>). Under rainfed condition, the crop growth was subject to water stress; under irrigation condition, there was no effect of water stress on crop growth. The gridded yield (*Yld*) was aggregated from rained and irrigated yield as follow [7]:

$Yld=\frac{{Yld}_{r}\times{Area}_{r}+{Yld}_{i}\times{Area}_{i}}{{Area}_{r}+{Area}_{i}}$ (1)

respectively. ${Area}_{r}$ and ${Area}_{i}$ are the rainfed and irrigated harvest area per crop in a grid cell, respectively. The rainfed and irrigated harvest areas data were obtained from MIRCA2000 [8]. The national yield was then aggregated from the gridded yield and weighted according to the crop-specific total harvest area.

Irrigation contribution (*IC*) in a country (c) was estimated as follow [7]:

*IC* = $\frac{{Yld}_{i,c}*{Area}_{i,c}}{{Yld}_{i,c}*{Area}_{i,c}+{Yld}_{r},c*{Area}_{r,c}}$*100% (2)

where ${Yld}_{i,c}$ and ${Yld}_{r,c}$ are the irrigated and rainfed yields for a country, respectively; ${Area}_{i,c}$ and ${Area}_{r,c}$ are the total irrigated and rainfed harvest areas for a country, respectively.

To analyze the sensitivity of yield variation relative to the expansion of the irrigated area in future, we assumed that a given fraction (*f*) (in this study, 10%, 15%, 20%, 25%, 30%, and 35%) of the area of rainfed cropland was converted into irrigated cropland. For each case, the corresponding yield for each country was recalculated as follows:

$Yld,c=\frac{{Yld}_{r,c}\times{(Area}_{r,c}-{f*Area}_{r,c})+{Yld}_{i,c}\times{(Area}_{i,c}+{f*Area}_{r,c})}{{Area}_{r,c}+{Area}_{i,c}}$ (3)


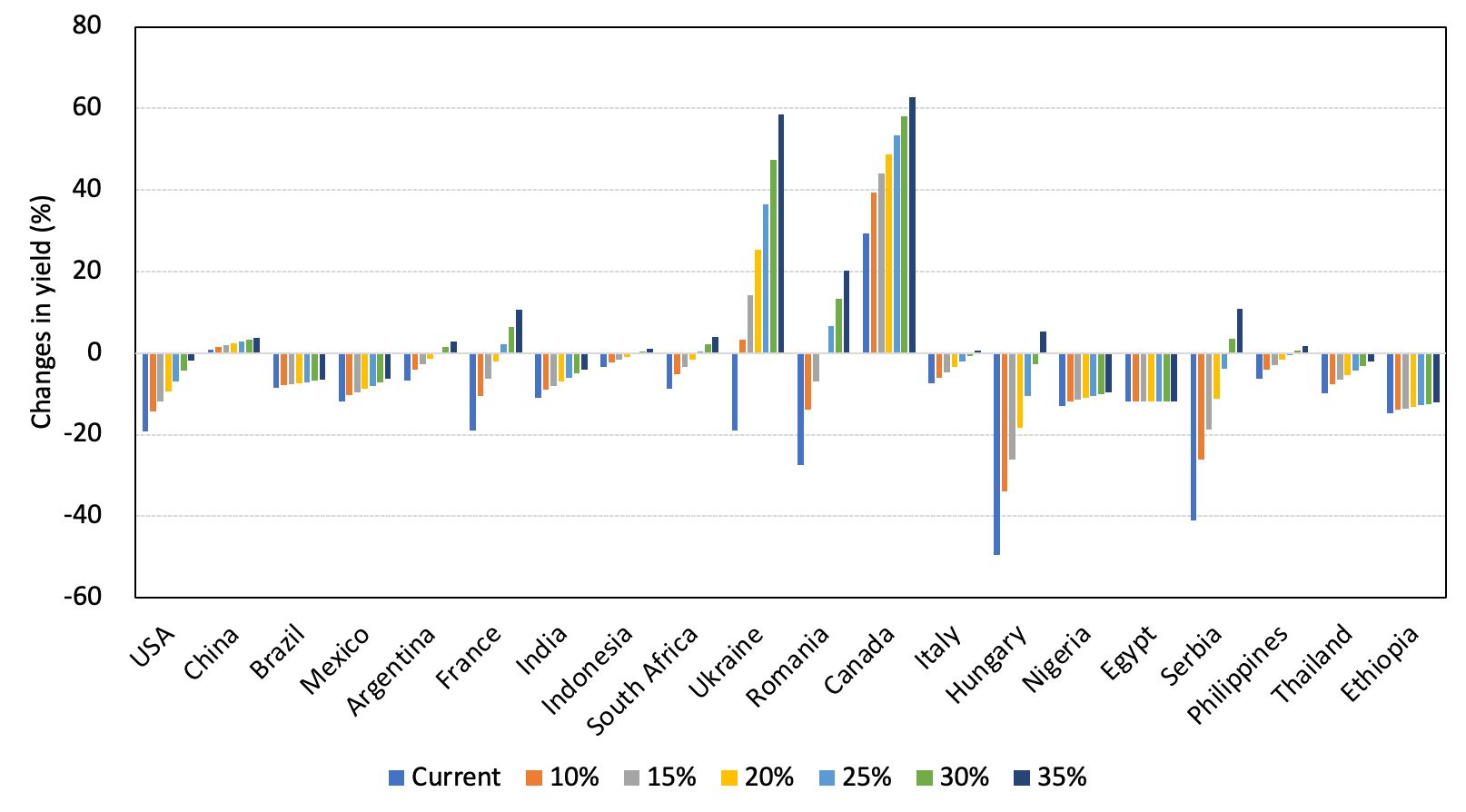


Fig. S1 Sensitivity of the changes in maize yield under different irrigation conditions. The changes represented the difference in the yield for the future period (2070–2099) relative to the historical period (1986–2015). In the “current” condition, the irrigation area in the future is remained the same as that in the historical period. In the remaining conditions, 10%, 15%, 20%, 25%, 30%, and 35% of the current rainfed area was converted into irrigated area for each country.

Table S1 Top 20 maize-, wheat-, and rice-producing countries based on Food and Agriculture Organization average production data (1986–2015).

| Rank | Maize | Wheat | Rice |
| --- | --- | --- | --- |
| 1 | USA | China | China |
| 2 | China | India | India |
| 3 | Brazil | USA | Indonesia |
| 4 | Mexico | Russia | Bangladesh |
| 5 | Argentina | France | Vietnam |
| 6 | France | Canada | Thailand |
| 7 | India | Germany | Myanmar |
| 8 | Indonesia | Turkey | Philippines |
| 9 | South Africa | Australia | Japan |
| 10 | Ukraine | Pakistan | Brazil |
| 11 | Romania | Ukraine | USA |
| 12 | Canada | UK | Pakistan |
| 13 | Italy | Argentina | South Korea |
| 14 | Hungary | Kazakhstan | Egypt |
| 15 | Nigeria | Iran | Cambodia |
| 16 | Egypt | Poland | Nepal |
| 17 | Serbia | Italy | Nigeria |
| 18 | Philippines | Egypt | Madagascar |
| 19 | Thailand | Romania | Sri Lanka |
| 20 | Ethiopia | Spain | North Korea |

Table S2 Maize yield (*Yld*), harvest area (*Area*), and production (*Pro*) in the top 20 maize-producing countries. Values are rounded to one decimal place. The subscripts *i*, *r*, and *t* indicate the values under irrigated, rainfed, and total conditions, respectively.

| Country | *Yld_i_*  (t/ha) | *Yld_r_*  (t/ha) | *Area_i_*  (Mha) | *Area_r_*  (Mha) | *Pro_i_*  (Mt) | *Pro_r_*  (Mt) | *Pro_t_*  (Mt) | *IC*  (%) |
| --- | --- | --- | --- | --- | --- | --- | --- | --- |
| USA | 10.1 | 6.7 | 4.8 | 27.2 | 48.4 | 183.2 | 231.6 | 20.9 |
| China | 4.6 | 3.9 | 12.8 | 11.9 | 59.0 | 46.2 | 105.1 | 56.1 |
| Brazil | 3.1 | 3.0 | 0.1 | 11.2 | 0.3 | 33.8 | 34.1 | 0.9 |
| Mexico | 3.0 | 2.4 | 1.6 | 5.8 | 4.8 | 14.1 | 18.9 | 25.4 |
| Argentina | 6.3 | 4.8 | 0.1 | 3.4 | 0.7 | 16.4 | 17.1 | 3.8 |
| France | 10.9 | 5.0 | 0.9 | 2.2 | 9.4 | 11.2 | 20.6 | 45.6 |
| India | 2.3 | 1.8 | 1.5 | 5.1 | 3.5 | 9.0 | 12.5 | 28.3 |
| Indonesia | 3.2 | 2.8 | 0.3 | 2.6 | 1.0 | 7.3 | 8.3 | 12.5 |
| South Africa | 3.8 | 2.7 | 0.1 | 2.9 | 0.5 | 7.9 | 8.4 | 5.7 |
| Ukraine | 8.2 | 2.3 | 0.2 | 3.2 | 1.5 | 7.2 | 8.7 | 17.6 |
| Romania | 5.4 | 2.2 | 0.1 | 2.9 | 0.6 | 6.5 | 7.1 | 8.0 |
| Canada | 9.3 | 5.3 | 0.0 | 1.4 | 0.3 | 7.4 | 7.7 | 4.4 |
| Italy | 9.7 | 4.5 | 0.8 | 0.5 | 7.5 | 2.3 | 9.9 | 76.4 |
| Hungary | 10.1 | 4.7 | 0.0 | 1.2 | 0.1 | 5.8 | 5.9 | 1.5 |
| Nigeria | 1.7 | 1.5 | 0.0 | 3.6 | 0.0 | 5.6 | 5.6 | 0.6 |
| Egypt | 6.6 | 0.0 | 0.8 | 0.0 | 5.4 | 0.0 | 5.4 | 100.0 |
| Serbia | 3.8 | 1.7 | 0.0 | 1.0 | 0.0 | 1.6 | 1.6 | 1.5 |
| Philippines | 0.0 | 1.7 | 0.0 | 2.4 | 0.0 | 4.0 | 4.0 | 0.0 |
| Thailand | 3.8 | 3.2 | 0.0 | 1.2 | 0.0 | 3.7 | 3.7 | 0.0 |
| Ethiopia | 1.4 | 1.3 | 0.1 | 1.1 | 0.1 | 1.4 | 1.5 | 8.8 |

Table S3 Wheat yield (*Yld*), harvest area (*Area*), and production (*Pro*) in the top 20 wheat-producing countries. Values are rounded to one decimal place. The subscripts *i*, *r*, and *t* indicate the values under irrigated, rainfed, and total conditions, respectively.

| Country | *Yld_i_*  (t/ha) | *Yld_r_*  (t/ha) | *Area_i_*  (Mha) | *Area_r_*  (Mha) | *Pro_i_*  (Mt) | *Pro_r_*  (Mt) | *Pro_t_*  (Mt) | *IC*  (%) |
| --- | --- | --- | --- | --- | --- | --- | --- | --- |
| China | 4.0 | 3.0 | 21.6 | 9.3 | 85.4 | 28.3 | 113.8 | 75.1 |
| India | 2.6 | 1.1 | 22.9 | 3.7 | 60.1 | 4.1 | 64.2 | 93.6 |
| USA | 5.0 | 2.4 | 1.2 | 21.2 | 6.0 | 50.2 | 56.2 | 10.7 |
| Russia | 3.4 | 1.4 | 0.5 | 19.1 | 1.8 | 26.4 | 28.2 | 6.2 |
| France | 7.8 | 6.1 | 0.0 | 4.7 | 0.3 | 28.9 | 29.1 | 0.9 |
| Canada | 5.2 | 2.2 | 0.1 | 10.5 | 0.5 | 23.2 | 23.7 | 1.9 |
| Germany | 7.6 | 6.3 | 0.0 | 2.5 | 0.0 | 15.9 | 15.9 | 0.1 |
| Turkey | 3.3 | 1.9 | 1.0 | 7.9 | 3.4 | 14.6 | 18.1 | 19.0 |
| Australia | 2.6 | 1.5 | 0.1 | 11.6 | 0.3 | 17.2 | 17.4 | 1.5 |
| Pakistan | 2.2 | 1.2 | 7.9 | 0.4 | 17.4 | 0.5 | 17.9 | 97.4 |
| Ukraine | 3.7 | 2.1 | 0.1 | 5.4 | 0.5 | 11.2 | 11.8 | 4.7 |
| UK | 8.3 | 6.5 | 0.0 | 1.8 | 0.0 | 11.4 | 11.4 | 0.3 |
| Argentina | 2.6 | 2.1 | 0.1 | 6.0 | 0.2 | 12.5 | 12.7 | 1.4 |
| Kazakhstan | 3.2 | 0.7 | 0.1 | 9.3 | 0.4 | 6.3 | 6.8 | 6.6 |
| Iran | 2.2 | 1.1 | 2.2 | 3.3 | 4.9 | 3.6 | 8.4 | 57.9 |
| Poland | 4.0 | 3.5 | 0.0 | 2.5 | 0.0 | 8.7 | 8.7 | 0.2 |
| Italy | 3.6 | 2.9 | 0.1 | 1.6 | 0.4 | 4.5 | 4.9 | 7.5 |
| Egypt | 5.1 | 3.2 | 1.0 | 0.0 | 5.2 | 0.0 | 5.2 | 99.9 |
| Romania | 3.2 | 2.3 | 0.1 | 1.9 | 0.3 | 4.5 | 4.8 | 7.1 |
| Spain | 3.2 | 2.3 | 0.2 | 2.1 | 0.5 | 4.6 | 5.1 | 9.9 |

Table S4 Rice yield (*Yld*), harvest area (*Area*), and production (*Pro*) in the top 20 rice-producing countries. Values are rounded to one decimal place. The subscripts *i*, *r*, and *t* indicate the values under irrigated, rainfed, and total conditions, respectively.

| Country | *Yld_i_*  (t/ha) | *Yld_r_*  (t/ha) | *Area_i_*  (Mha) | *Area_r_*  (Mha) | *Pro_i_*  (Mt) | *Pro_r_*  (Mt) | *Pro_t_*  (Mt) | *IC*  (%) |
| --- | --- | --- | --- | --- | --- | --- | --- | --- |
| China | 5.4 | 5.2 | 37.7 | 4.7 | 204.8 | 24.4 | 229.2 | 89.4 |
| India | 2.7 | 2.1 | 23.9 | 20.2 | 65.7 | 42.3 | 108.0 | 60.8 |
| Indonesia | 4.2 | 3.7 | 5.5 | 5.3 | 23.2 | 19.6 | 42.9 | 54.2 |
| Bangladesh | 3.4 | 2.6 | 5.3 | 5.1 | 17.9 | 13.4 | 31.3 | 57.2 |
| Vietnam | 4.0 | 3.5 | 4.4 | 2.3 | 17.4 | 8.0 | 25.3 | 68.5 |
| Thailand | 2.6 | 2.3 | 4.5 | 5.3 | 11.5 | 12.0 | 23.5 | 48.8 |
| Myanmar | 3.5 | 2.7 | 1.9 | 4.2 | 6.6 | 11.3 | 17.9 | 36.9 |
| Philippines | 2.8 | 2.5 | 1.6 | 1.9 | 4.6 | 4.8 | 9.4 | 48.5 |
| Japan | 5.4 | 0.0 | 1.6 | 0.0 | 8.8 | 0.0 | 8.8 | 100.0 |
| Brazil | 3.1 | 2.4 | 0.9 | 2.1 | 2.7 | 5.0 | 7.7 | 34.5 |
| USA | 6.4 | 4.9 | 1.3 | 0.0 | 8.2 | 0.2 | 8.4 | 97.7 |
| Pakistan | 2.7 | 0.6 | 3.0 | 0.0 | 8.0 | 0.0 | 8.0 | 100.0 |
| South Korea | 5.6 | 5.5 | 0.6 | 0.4 | 3.5 | 2.3 | 5.9 | 60.3 |
| Egypt | 7.5 | 0.0 | 0.6 | 0.0 | 4.9 | 0.0 | 4.9 | 100.0 |
| Cambodia | 1.9 | 1.7 | 0.3 | 1.8 | 0.7 | 3.1 | 3.7 | 17.4 |
| Nepal | 2.5 | 1.9 | 0.4 | 1.0 | 1.1 | 1.8 | 2.9 | 37.5 |
| Nigeria | 1.5 | 1.5 | 0.0 | 2.0 | 0.0 | 3.0 | 3.0 | 0.4 |
| Madagascar | 2.1 | 0.0 | 1.0 | 0.0 | 2.1 | 0.0 | 2.1 | 100.0 |
| Sri Lanka | 3.2 | 2.8 | 0.6 | 0.2 | 1.9 | 0.4 | 2.3 | 81.0 |
| North Korea | 3.7 | 3.7 | 0.4 | 0.2 | 1.4 | 0.6 | 2.0 | 70.4 |

**Reference**

1. Hanasaki N, Kanae S, Oki T et al. Hydrol Earth Syst Sci 2008a; **12**: 1007–1025.
2. Hanasaki N, Yoshikawa S, Pokhrel Y et al. Hydrol Earth Syst Sci 2018; **22**: 789–817.
3. Hanasaki N, Kanae S, Oki T et al. Hydrol Earth Syst Sci 2008b; **12**: 1027–1037.
4. Hanasaki N, Kanae S, Oki T. J Hydrol 2006; **327**: 22–41.
5. Hanasaki N, Inuzuka T, Kanae S et al. J Hydrol 2010; **384**: 232–244.
6. Ai Z, Hanasaki N, Heck V et al. Nat Sustainability 2021; **4**: 884–891.
7. Ai Z, Hanasaki N. Geosci Model Dev 2023; **16**: 3275–3290.
8. Portmann F, Siebert S, Döll P. Global Biogeochem Cy 2010; **24**, 1–24.
9. Müller C, Elliott J, Chryssanthacopoulos J et al. Geosci Model Dev 2017; **10**, 1403–1422.
10. Jägermeyr J, Müller C, Ruane AC et al. Nat. Food 2021; **2**, 873–885.
